# Understanding Intentions to Discuss Long-Acting Injectable Pre-Exposure Prophylaxis with Healthcare Providers Among Black and Hispanic Gay and Bisexual Men in Texas

**DOI:** 10.1177/23259582251336662

**Published:** 2025-04-30

**Authors:** Chukwuemeka N Okafor, Jin Yoon, Angela Heads, Joy Schmitz

**Affiliations:** 1 Department of Medicine, Division of Infectious Diseases, Long School of Medicine, University of Texas Health Science Center San Antonio, San Antonio, Texas, USA; 2 Be Well Institute for Substance Use and Related Disorders, Long School of Medicine, University of Texas Health Science Center San Antonio, San Antonio, Texas, USA; 3 McGovern Medical School, University of Texas Health Science Center, Houston, TX, USA

**Keywords:** HIV prevention, long-acting injectable PrEP, black and Hispanic gay men, healthcare provider discussions, stigma, discrimination, theory of planned behavior

## Abstract

We examined factors influencing the intention of Black and Hispanic gay and bisexual men aged 18-34 years in Texas to discuss starting long-acting injectable pre-exposure prophylaxis (LAI-PrEP) with healthcare providers. Participants were recruited through geosocial apps and community locations, completed online surveys measuring attitudes, subjective norms, perceived behavioral control (Theory of Planned Behavior), internalized homophobia, medical mistrust, HIV risk, and medical mistrust. Among the final sample (*N* = 190), 63.5% intended to discuss LAI-PrEP. Poisson regression models indicated that higher attitudinal concerns [adjusted prevalence ratio (aPR): 0.80, 95% confidence interval (CI): 0.70, 0.92; *P* < 0.01) and higher medical mistrust (aPR: 0.98, 95% CI: 0.97, 0.99; *P* = 0.01) were linked to lower prevalence of intentions. Seeing a doctor in the past 12 months was associated with higher prevalence of discussing LAI-PrEP (aPR: 1.46, 95% CI: 1.00, 2.13; *P* = 0.05). Addressing concerns and reducing discrimination are crucial for improving LAI-PrEP uptake in this population.

## Plain Language Summary

*Understanding why Black and Hispanic gay and bisexual men in Texas choose to talk or not talk to their doctors about long-acting injectable PrEP, a medicine that helps prevent HIV*

In the U.S., young Black and Hispanic gay and bisexual men are disproportionately affected by HIV, especially in Southern states like Texas. These groups face high rates of new HIV infections, yet they often have less access to effective prevention options like PrEP (pre-exposure prophylaxis), which can help prevent HIV. Texas, in particular, has low rates of PrEP use, despite having some of the highest numbers of new HIV cases.

A new form of PrEP, known as long-acting injectable PrEP (LAI-PrEP), offers a different approach to HIV prevention. Instead of taking a daily pill, LAI-PrEP involves getting an injection every 2 months, which could make it easier for some people to stay protected. While many studies have looked at how willing people are to use LAI-PrEP, fewer have explored the crucial first step: whether individuals intend to talk to their healthcare providers about starting this preventive treatment.

This study focuses on understanding the intentions of Black and Hispanic gay and bisexual men in Texas to discuss LAI-PrEP with their doctors. It's important because starting a conversation with a healthcare provider is a key step in accessing HIV prevention tools. The study will examine factors like attitudes towards LAI-PrEP, perceived social pressures, and confidence in discussing PrEP with a doctor. It will also look at how experiences of discrimination, medical mistrust, and internalized homophobia might affect these intentions.

By understanding these factors, we can better address the barriers that prevent people from accessing LAI-PrEP and tailor interventions to support individuals in taking this important step toward HIV prevention.

## Introduction

In the United States (U.S), Black and Hispanic gay and bisexual men (GBM) aged 18-34 years are disproportionately affected by HIV and bear an overwhelming burden of new infections each year.^1^ In 2020, GBM accounted for 68% of new HIV diagnoses in the U.S, of which over a third of these new diagnoses were among Black/African American and Hispanic/Latino. Moreover, over half of new HIV diagnoses in the U.S occurred among those 18-34 years of age.^1^ Furthermore, the HIV epidemic in the U.S is concentrated in the Southern U.S, with over half of new HIV diagnoses occurring in Southern states.^1^ Among Southern states, Texas had the second-largest number of new HIV diagnoses in the South^2–4^ with Black/African American and Hispanic persons accounting for 42.8 and 14.7 new cases per 100 000 population in 2019, respectively—representing the two highest racial/ethnic groups.^3^ Therefore, there is an urgent need for targeted HIV prevention research among young, minority GBM in the Southern U.S.

Despite the availability of effective HIV prevention options such as daily oral pre-exposure prophylaxis (PrEP), disparities in access and uptake persist.^5^ For instance, Texas ranked 40th across U.S states in PrEP uptake in 2018,^6^ yet ranked second among U.S states for new HIV diagnoses in 2022.^4^ Long-acting injectable (LAI) cabotegravir HIV PrEP or LAI-PrEP (ie, *Apretude*) is a groundbreaking advancement in HIV prevention.^7^ Treatment with LAI-PrEP begins with two initial injections 1 month apart, followed by injections every 2 months.^8^ This approach offers an alternative to daily oral PrEP, potentially overcoming the difficulties with daily dosing schedule required with oral PrEP.^9^ Studies have documented high interest and willingness to use LAI-PrEP among GBM.^10–12^ Whereas most prior studies have focused on understanding the factors influencing willingness to use LAI-PrEP,^10–13^ few have addressed early steps in this process, such as intentions to discuss starting this preventive option with healthcare providers.

The intention to discuss starting LAI-PrEP with healthcare providers is a critical first step in the process of LAI-PrEP uptake, given that among PrEP eligible sexual and gender minority persons who spoke with a medical provider about PrEP indicated that they would initiate PrEP.^14^ While the willingness to use LAI-PrEP reflects an individual's readiness to consider this preventive option, it does not capture the complexities of initiating a conversation with healthcare providers. Evaluating intentions to discuss LAI-PrEP can uncover specific obstacles such as fear of stigma, discrimination, lack of cultural concordance with PrEP providers or negative past experiences with healthcare systems or healthcare providers,^15–17^ which may impact whether individuals follow through with their willingness to initiate HIV prevention tools such as LAI-PrEP. By focusing on these initial steps, we can more effectively address barriers to engagement and tailor interventions to help individuals take the next step toward utilizing LAI-PrEP successfully.

There are myriad factors that could influence Black and Hispanic gay and bisexual men's intentions to discuss starting LAI-PrEP with their healthcare providers. Discrimination based on racial, ethnic and sexual minority status creates significant barriers when considering PrEP.^18^ Healthcare systems in the U.S have a history of medical mistreatment of African Americans, contributing to a legacy of mistrust. As such, negative experiences with healthcare providers and the healthcare system can foster medical mistrust regarding treatments and HIV prevention methods.^19,20^ Consequently, perceived and/or experienced discrimination, particularly among gay and bisexual men, could negatively impact intentions to discuss LAI-PrEP with healthcare providers. Additionally, social support networks that minimize the importance of HIV prevention or LAI-PrEP can reduce motivation to seek or discuss HIV prevention options like LAI-PrEP with healthcare providers, due to fears of negative reactions from their community.^21,22^ Internalized homophobia characterized by internalized negative attitudes toward gay or bisexual people and discomfort with disclosing their sexual orientation status to others can also negatively influence intentions to discuss LAI-PrEP with healthcare providers. Consequently, prior studies have shown internalized homophobia to be associated with reduced PrEP uptake among gay and bisexual men.^23,24^ There is a notable lack of studies specifically examining these and other psychosocial factors that could potentially influence intentions to discuss LAI-PrEP with healthcare providers.

To address this gap in the literature, we employed the Theory of Planned Behavior (TPB) to assess factors influencing intentions to discuss LAI-PrEP with a healthcare provider. The TPB is a well-established behavioral model that seeks to explain how individuals form intentions to engage in a specific health behavior (eg, PrEP use).^25,26^ It posits that intentions to engage in a specific behavior are influenced by three primary constructs: *attitudes* (eg, positive or negative evaluation of performing the behavior), *subjective norms* (perceived social pressures to perform or not perform the behavior), and *perceived behavioral control* (an individual's perceptions of their ability to execute the behavior).^25,26^ Previous research has utilized the TPB to understand PrEP acceptability and uptake among gay and bisexual men^27^ and other populations that could benefit from PrEP.^28–30^ These studies have generally found that negative attitudes, negative subjective norms, and low perceived behavioral control are associated with intentions and willingness to uptake PrEP. However, few studies have utilized the TPB to assess factors influencing intentions to discuss starting LAI-PrEP with a healthcare provider.

The objective of this study was to examine TPB constructs associated with intentions to discuss starting LAI-PrEP with healthcare providers among a sample of Black and Hispanic gay and bisexual men in Texas. In addition, we also assessed key psychosocial factors that could negatively influence intentions to discuss LAI-PrEP with healthcare providers including internalized homophobia, medical mistrust, racial and sexual discrimination, and HIV risk perceptions. We hypothesized that TPB constructs (attitudes, subjective norms, and perceived behavioral control) and these key psychosocial factors will be associated with intentions to discuss LAI-PrEP with healthcare providers.

## Methods

### Recruitment and Eligibility Criteria

Participants were recruited (between May 2023 and March 2024) in Texas using targeted strategies. Study advertisements were posted on geosocial apps such as Jack’D, Grindr, and Scruff, with geographic targeting to Texas. Additionally, study flyers were distributed at community-based organizations, sexual health clinics, coffee shops, and other locations in San Antonio and Houston. Interested individuals could access the screening questionnaire via a QR code on the study flyer or a study link, which was administered through Qualtrics. The screening questionnaire assessed eligibility based on predefined criteria, including: (1) 18-34 years of age, (2) HIV-negative or unknown HIV status, (3) Black/African American or Hispanic/Latino, (4) assigned male sex at birth, (5) identifying as gay or bisexual, (6) having anal sex with another man in the past 6 months, and (7) having anal sex with 2 + different male partners in the past 6 months. Individuals who met all eligibility criteria were automatically advanced to the consent form, and those who consented proceeded directly to the survey. Participants who completed the survey received a $25 electronic gift card via email. All study procedures were approved by UT Health San Antonio Institutional Review Board.

### Validity of Survey Responses

Survey responders were screened for validity using several approaches. Qualtrics fraud detection tools were used to detect multiple submissions (ie, duplicate responses) and bots (using Google's reCAPTCHA technology), which were subsequently excluded from further analysis. Additionally, respondents outside of Texas were flagged and removed using the GeoIP location tool in Qualtrics, which uses the latitude and longitude coordinates of the IP addresses of respondents to estimate their location. Finally, survey responses with infeasibly short completion times (less than 10 min) were excluded from further analysis.

### Analytic Sample

Two hundred fifty-eight of 1457 individuals who provided consent met eligibility criteria and started the survey. Out of 258, 62 were excluded for not finishing the survey and 6 were excluded for infeasibly short completion times (less than 10 min). Therefore, the final analytic sample comprised *N* = 190 individuals.

## Measures

### Theory of Planned Behavior Constructs

We identified items measuring TPB constructs from published studies on PrEP attitudes and norms.^28,29,31^ For this study, we used three items to measure attitudes toward LAI-PrEP, three items to measure subjective norms, and five items to measure perceived behavioral control. All items are listed in Table 1. Participants were provided the following instructions for completing the questionnaire: “Select the answer that best describes your view regarding the PrEP shot.” Item response options were on a 5-point Likert scale, ranging from 1 (Not at all concerned) to 5 (Extremely concerned). We created a mean score for each TPB construct. Cronbach *α* was 0.89.

**Table 1.** Participant Characteristics by Intention to Discuss LAI-PrEP with a Doctor.

			Intend to discuss with doctor about LAI-PrEP?				
	Overall		Yes (*N* = 106)		No (*N* = 61)		
	*N*	%	*n*	%	*n*	%	*P*
**Age (mean, SD)**	28.1	4.1	28	4.2	28	3.8	0.52
**Race/ethnicity**							
Non-Hispanic Black	97	51.3	48	45.7	30	49.2	0.71
Hispanic White	49	25.9	33	31.4	15	24.6	
Hispanic Black	15	7.9	10	9.5	5	8.2	
Hispanic other race	28	14.8	14	13.3	11	18.0	
**Sexual orientation**							
Gay	149	78.4	79	74.5	48	78.7	0.54
Bisexual	41	21.6	27	25.5	13	21.3	
**Education**							
Some college or less	71	41.0	43	41.3	22	39.3	0.81
Associate degree	31	17.9	18	17.3	12	21.4	
College or more	71	41.0	43	41.3	22	39.3	
**Employment**							
Yes	154	81.9	86	81.1	49	83.1	0.75
No	34	18.1	20	18.9	10	16.9	
**Health Insurance**							
Yes	132	74.6	78	75.7	42	72.4	0.64
No	45	25.4	25	24.3	16	27.6	
**Seen doctor-past 12mo**							
Yes	141	78.8	92	86.8	42	73.7	0.03
No	38	21.2	14	13.2	15	26.3	
**Currently using oral PrEP**							
Yes	93	59.2	55	58.5	33	62.3	0.65
No	64	40.8	39	41.5	20	37.7	
**HIV risk perception**							
Zero/almost zero	63	33.2	31	29.2	21	34.4	0.68
Small	70	36.8	43	40.6	25	41.0	
Moderate-very large	57	30.0	32	30.2	15	24.6	
**Aware about LAI-PrEP**							
Yes	112	66.3	65	63.7	37	68.5	0.54
No	57	33.7	37	36.3	17	31.5	
**No of sexual partners (mean, SD)**	7.2	7.9	8.0	7.7	6.3	7.7	0.02
**Group-based medical mistrust scale (GBMMS, mean SD)**	33.6	8.3	32.4	8.7	35.5	8.0	0.02
GBMMS suspicion subscale	16.0	5.1	15.2	5.4	17.1	4.5	<0.01
GBMMS discrimination subscale	9.4	2.7	9.2	2.8	9.7	2.8	0.25
GBMMS lack of social support subscale	8.2	2.7	8.1	2.7	8.6	2.8	0.28
**Multidimensional scale perceived social support (MSPSS, mean SD)**	4.5	1.3	4.7	1.3	4.3	1.3	0.14
MSPSS family	4.5	1.6	4.7	1.6	4.1	1.7	0.62
MSPSS friends	4.3	1.5	4.4	1.6	4.3	1.5	0.39
MSPSS significant other	4.7	1.5	4.9	1.5	4.6	1.5	0.01
**Racial discrimination, mean SD**	3.3	2.7	3.1	2.9	3.4	2.3	0.23
**Sexual discrimination, mean SD**	3.3	2.8	3.0	3.0	3.4	2.7	0.21
**Internalized homophobia, mean SD**	32.5	8.4	32.8	9.0	32.8	7.3	0.98
**Theory of planned behavior constructs, mean SD**							
**Attitude**	2.1	1.2	2.4	1.0	2.8	1.0	0.02
I am concerned that the PrEP shot does not provide complete protection against HIV	2.5	1.3	2.4	1.2	2.6	1.3	0.34
I am concerned that the PrEP shot may have a bad interaction with other medications that I am taking.	2.2	1.4	2.1	1.3	2.4	1.6	0.34
I am concerned about the long-term effects of the PrEP shot on my health	2.8	1.4	2.6	1.3	3.2	1.4	<0.01
**Subjective norms**	2.5	1.1	2.5	1.1	2.6	1.0	0.52
I am concerned that people who know I am taking the PrEP shot may think I have HIV.	1.9	1.3	1.9	1.3	1.8	1.3	0.98
I am concerned that if people know I am taking the PrEP shot, they will want to know why I am taking it	2.1	1.4	2.1	1.4	1.9	1.3	0.47
I think people would judge me if I were taking the PrEP shot.	1.9	1.3	2.0	1.4	1.9	1.3	0.52
**Behavioral control**	2.5	1.1	2.5	1.1	2.6	1.0	0.55
I am concerned about how I will pay for the PrEP shot.	2.4	1.4	3.3	1.5	2.8	1.6	0.05
I am concerned about having to take the PrEP shot every two months.	2.4	1.4	2.3	1.4	2.6	1.5	0.16
I am concerned about injection site reactions of the PrEP shot.	2.4	1.4	2.4	1.4	2.4	1.4	0.79
I am concerned about having to come to the clinic regularly to take the PrEP shot.	2.2	1.5	2.3	1.4	2.4	1.4	0.75
I am concerned that I would not have control over starting or stopping the medication because it would be in my body for 8 weeks or longer after the injection.	2.4	1.4	2.0	1.4	2.4	1.6	0.07

*Note:* LAI-PrEP: long-acting injectable pre-exposure prophylaxis.

#### Intention to Discuss with Healthcare Provider About Getting LAI-PrEP

Participants were asked whether they intended to discuss getting “the PrEP shot” with a healthcare provider. Participants were first provided with the following description about the PrEP shot. “*Long-acting injectable Pre-exposure prophylaxis (Apretude/PrEP shot) is an injection or shot in the muscle of the butt taken every two months. Results from clinical studies showed that a PrEP shot was better in preventing people from getting HIV when compared to the PrEP taken as a pill every day. PrEP shot has been approved by the U.S Food and Drug Administration for HIV prevention.”* Participants were then asked: “Do you intend to discuss with your healthcare provider about getting the PrEP shot?” Response options were 1 = Yes, I will definitely discuss with my healthcare provider about the PrEP shot and 0 = No, I definitely will not discuss with my healthcare provider about the PrEP shot.

### Sociodemographic Characteristics

Participants self-reported their age (in years), race and ethnicity, sexual orientation, highest educational level attained, employment status, health insurance status, and whether or not they had seen a doctor in the past 12 months.

### Pre-Exposure Prophylaxis

Participants were asked if they have ever used oral PrEP with the following question “*Have you ever taken oral PrEP?”* with response options of “No” and “Yes.” Participants who responded with a “Yes” were further asked: “*Are you currently taking PrEP?”* with response options of “No” and “Yes.”

### HIV Risk Perception

Participants were asked a single question on their perceived risk of acquiring HIV with the following question: “I think my chances of getting infected with HIV are?” with response options of “Zero,” “Almost zero,” “Small,” “Moderate,” “Large,” and “Very large.”^32^

### Sexual Behaviors

Participants were asked how many different men they had anal sex with in the past 6 months.

### Medical Mistrust

The Group-Based Medical Mistrust Scale (GBMMS) is a 12-item scale used to measure distrust or suspicion towards healthcare institutions, healthcare providers, and the healthcare system as a whole.^33^ The GBMMS was developed to assess the extent to which individuals from racial and ethnic minority groups perceive healthcare institutions as trustworthy. Response options on the GBMMS were on a Likert scale ranging from 1 = Strongly disagree to 5 = Strongly agree. Mean scores were calculated for the full scale and the subscales: suspicion, discrimination, and lack of social support. Cronbach *α* was 0.80.

### Perceived Social Support

The Multidimensional Scale Perceived Social Support (MSPSS) was used to measure perceived social support across various sources.^34^ The MSPSS is a 12-item scale that assesses the adequacy of perceived social support from family, friends, and significant others. Response options on the MSPSS were on a 7-point Likert scale ranging from 1 = very strongly disagree to 7 = very strongly agree. Mean scores were calculated for the full scale and the subscales representing sources of social support: family, friends, and significant. Cronbach *α* was 0.89.

### Racial and Sexual Discrimination

We assessed how participants experienced dual discrimination based on both their sexual orientation and race and ethnicity.^35^ Each participant responded to 10 statements regarding discriminatory incidents over the past year related to each identity (0 = No, 1 = Yes). Examples include being treated with hostility by strangers due to race/ethnicity or having personal property damaged or stolen because of sexual identity. We then calculated a summed score for each discrimination scale. Cronbach *α* was 0.89 (for sexual discrimination) and 0.78 (for racial discrimination).

### Internalized Homophobia

Internalized homophobia was assessed with the Internalized Homophobia Scale,^36^ which uses a past 12-month reference period. Example items included: “I tried to stop being attracted to men in general”; “I felt alienated from myself because of being gay/bisexual”. The response options were (1) strongly disagree; (2) disagree; (3) neutral; (4) agree; (5) strongly agree; (6) don’t know/unsure; and (7) prefer not to say. Cronbach *α* was 0.86.

## Data Analysis

We calculated descriptive statistics for each variable for the entire sample, as well as stratified by whether participants *intended to discuss with healthcare provider about getting LAI-PrEP*. We used Chi-square or Fisher's exact tests or t-tests in bivariable comparisons between all variables with the intention to discuss LAI-PrEP with a doctor. In constructing our multivariable model, we retained all three TPB constructs alongside age and HIV risk perception in the model regardless of their statistical significance. We included other variables that showed a statistically significant association (*P* < 0.05) with the intention to discuss LAI-PrEP with a doctor in the bivariate analysis into the modified Poisson regression model with robust error variances.^37^ Missing data for variables were as follows: intention to discuss with healthcare provider at getting the PrEP shot (12%) and seen doctor in the past 12 months (5%), with all other variables missing at <1%. Missing data was addressed by multiple imputation using chained equations.^38^ Ten imputed data sets were generated for covariates, negative binomial regressions were conducted for each imputed dataset and the rate ratios were pooled according to Rubin's rules.^39^ Prevalence ratios and 95% confidence intervals (CIs) from the modified Poisson regression model are reported in Table 2.

**Table 2.** Prevalence Ratios of Factors Associated with Intention to Discuss LAI-PrEP with Doctor.

Characteristics	Prevalence ratios (95% confidence interval)	*P*-value
**Age**	1.01 (0.98, 1.04)	0.59
**HIV risk perception**		
Zero/almost zero	Reference	
Small	1.04 (0.80, 1.37)	0.73
Moderate-very large	1.19 (0.89, 1.59)	0.23
**Seen doctor in past 12 months**		
No	Reference	
Yes	1.46 (1.00, 2.13)	0.05
**Group-based medical mistrust scale**	0.98 (0.97, 0.99)	0.01
**Number of sexual partners**	1.01 (0.99, 1.01)	0.36
***Theory of planned behavior constructs***		
Attitude	0.80 (0.70, 0.92)	<0.01
Subjective norms	1.12 (0.98, 1.28)	0.08
Behavioral control	1.03 (0.88, 1.21)	0.36

**Note:** Lai-PrEP, long-acting injectable pre-exposure prophylaxis.

## Results

### Sample Characteristics

The study comprised a total of 190 participants, with a mean age of 28.1 years (SD = 4.1). The majority identified as Non-Hispanic Black (51.3%), followed by Hispanic White (25.9%), Hispanic Black (7.9%), and Hispanic of other races (14.8%). Most participants identified as gay (78.4%), with the remaining identifying as Bisexual (21.6%). Education levels varied among participants, with 41.0% having completed some college or less, 17.9% holding an associate degree, and another 41.0% having completed college or more. Most participants reported having health insurance (74.6%), and 78.8% had visited a doctor in the past 12 months. Participants’ mean number of sexual partners in the past 6 months was 7.2 (SD = 7.9). The majority of participants (63.5%) endorsed that they intended to discuss getting the PrEP shot with their healthcare provider.

### Bivariable Comparisons

Participants who had seen a doctor in the past 12 months were significantly more likely to report an intention to discuss with healthcare provider about getting LAI-PrEP compared to those who had not seen a doctor in the past 12 months (86.8% vs 13.2%, *P* = 0.03). The mean number of sexual partners in the past 6 months was significantly higher among those who indicated an intention to discuss with healthcare provider about getting LAI-PrEP (mean = 8.0) compared to those who did not indicate such an intention (mean = 6.3; *P* = 0.02). Mean score on the overall GBMMS was significantly lower among those who indicated an intention to discuss with healthcare provider about getting LAI-PrEP compared to those who did not (mean = 32.4 vs 35.5; *P* = 0.02). Finally, the mean score on attitudes regarding LAI-PrEP was significantly lower among those who indicated an intention to discuss with healthcare provider about getting LAI-PrEP compared to those who did not intend to discuss it (mean = 2.4 vs 2.8; *P* ≤ 0.02).

### Multivariable Model

In the final multivariable model, of the three TPB constructs, only attitudes were statistically significantly associated with prevalence of intention to discuss with healthcare provider about getting LAI-PrEP. Specifically, respondents with higher attitudinal concerns about LAI-PrEP had a lower prevalence of discussing LAI-PrEP with healthcare providers [adjusted prevalence ratio: 0.80, 95% CI: 0.70, 0.92, *P* < 0.01; Table 2]. Seeing a doctor in the past 12 months was associated with a higher prevalence of discussing LAI-PrEP with a healthcare provider, while higher levels of medical mistrust, as measured by the GBMMS, were associated with a lower prevalence of intending to have this discussion (Table 2).

## Discussion

The majority of participants in this study of Black and Hispanic gay and bisexual men aged 18-34 years in Texas expressed an intention to discuss getting the LAI-PrEP shot with their healthcare provider, highlighting a positive inclination towards this novel form of HIV prevention. This finding aligns with previous studies indicating a high interest in LAI-PrEP among this demographic group (Roth et al, 2019; Schnarrs et al, 2018; Tran et al, 2021).

Interestingly, attitudes towards LAI-PrEP were the only TPB construct significantly associated with the intention to discuss starting LAI-PrEP with a healthcare provider. Higher levels of attitudinal concerns were correlated with a lower prevalence of intending reduced prevalence of intention to discuss starting LAI-PrEP with a healthcare provider. This finding is consistent with other studies which utilized the TPB to understand PrEP acceptability and uptake. These studies have found that negative attitudes toward PrEP are associated with lower willingness and acceptability of PrEP among gay and bisexual men^27^ and other populations that could benefit from PrEP.^28–30^ In the current study, concerns about the long-term health effects of LAI-PrEP emerged as the key item within the attitudes construct from the TPB, that was significantly associated with lower intentions to discuss starting LAI-PrEP with a healthcare provider. Taken together, these findings underscore the importance of addressing specific concerns, particularly health concerns about LAI-PrEP, through targeted education and counseling strategies tailored to this population.

Our study also found that higher levels of medical mistrust from healthcare providers and the healthcare system were associated with lower prevalence of intention to discuss starting LAI-PrEP with healthcare providers. Our findings align with previous research highlighting the detrimental effects of medical mistrust and perceived discrimination on access to and uptake of PrEP^19,40–42^ and other HIV prevention services.^43^ Medical mistrust often arises from historical and ongoing discriminatory practices within healthcare settings, particularly those targeting minoritized populations. Racial, ethnic, and sexual minority groups, including Black and Hispanic GBM, have experienced disparities in healthcare access and quality due to discriminatory practices and biases within healthcare settings (Bogart et al, 2011). These experiences of discrimination can erode trust in healthcare providers and institutions, leading to reluctance in taking necessary steps to seek LAI-PrEP such as discussing with their healthcare providers about LAI-PrEP. Therefore, addressing discriminatory practices within healthcare settings is essential to promoting trust and engagement in HIV prevention services such as LAI-PrEP among Black and Hispanic GBM. Healthcare providers and institutions must implement strategies to mitigate discrimination, such as cultural competence training, and inclusive policies. These efforts are crucial for creating welcoming and affirming environments where individuals feel safe to discuss sensitive topics, such as sexual health and HIV prevention.

Additionally, other structural and systemic factors, such as policy restrictions and insurance coverage disparities play a significant tole in shaping access to healthcare and PrEP related discussing with medical providers in the U.S South. Texas, for example, has the largest number of uninsured residents among all 50 U.S states,^44^ largely due to the state's decision not to expand Medicaid, which limits healthcare access to persons with low income. In the current study, over a quarter of participants reported being uninsured, which is higher than most national estimates for sexual minority adults (18+ years), typically ranging from 3% to 14%,^45–48^ though at least one study has reported an uninsured prevalence as high as 27%.^14^ This lack of insurance coverage impedes healthcare access and reduces opportunities for PrEP discussions. Consistent with this, our findings indicate that individuals in the current study who had not seen a doctor in the past 12 months have lower prevalence of intention to discuss starting LAI-PrEP with healthcare providers. Addressing these barriers through expanded insurance coverage, and policy-level interventions is crucial for improving LAI-PrEP uptake among Black and Hispanic gay and bisexual men.

The implications of our findings extend to both research and clinical practice. Clinically, healthcare providers should be equipped with resources and training to effectively communicate the benefits and risks of LAI-PrEP, thereby addressing patient concerns and enhancing LAI-PrEP uptake. However, it is important to acknowledge that providers often face significant time constraints during visits, which can make addressing multiple concerns—such as explaining different PrEP options, and attending to other patient issues—challenging.^49,50^ Structural solutions, such as redefining clinic staff roles to include additional support for HIV prevention and counseling, incorporating peer navigators for HIV prevention education, and utilizing clinical decision support tools, such as reminders and prompts within the EHR to guide providers through PrEP screening and management, could help mitigate these challenges and optimize patient-provider interactions,^51,52^ could help mitigate these challenges and optimize patient-provider interactions. Moreover, strategies aimed at reducing perceived discrimination within healthcare settings are imperative, as our study identified lower prevalence of intention to discuss starting LAI-PrEP among individuals reporting higher levels of perceived discrimination. Furthermore, our study underscores the need for tailored interventions that consider the unique psychosocial factors influencing PrEP decision-making among Black and Hispanic gay and bisexual men. Interventions should integrate cultural competence and sensitivity to enhance trust and engagement within healthcare encounters, thereby promoting equitable access to LAI-PrEP.

Despite the significance of our findings, several limitations should be considered. First, the primary outcome of our study, intentions to discuss getting PrEP with a healthcare provider did not include a specific time frame, which may have made the assessment less precise. This lack of specificity could affect the interpretation of participants’ intentions, as their willingness to discuss LAI-PrEP with their healthcare providers could vary over different periods. Relatedly, all measures in our study were obtained via self-report which may introduce potential biases such as social desirability bias. Participants may have overreported socially desirable behaviors, such as intentions to discuss LAI-PrEP, or underreported stigmatized behaviors, such as sexual risk-taking. Second, some of the TPB constructs in this study were measured with limited items and may not capture broad attitudes, norms and behavioral control indicators. Measuring psychosocial constructs with few items can lead to lower internal consistency, making it difficult to accurately capture the construct. Although we note that the Cronbach *α* for these items was acceptable. Our recruitment strategy, which included online advertisements and community and clinic-based flyers, may have introduced selection bias. Individuals who are more engaged with digital platforms or specific community locations may differ systematically from those who are not. Additionally, our study's inclusion criteria of English proficiency may have excluded a substantial portion of the Spanish-speaking population, which represents a significant demographic group within Texas. These factors potentially limit the generalizability of our study findings. Additionally, while we restricted participation to individuals with Texas-based IP addresses, we cannot entirely rule out the possibility of virtual private network or VPN use, which may have affected geographic data. Future research may consider incorporating tools or screening measures to identify VPN usage and further ensure geographic accuracy*.*

In conclusion, this study highlights the significant factors influencing the intention to discuss starting LAI-PrEP among Black and Hispanic gay and bisexual men in Texas. The findings underscore that while there is a notable interest in discussing LAI-PrEP, concerns related to the PrEP shot and perceived discrimination within healthcare settings are critical barriers to initiating this medication. Specifically, negative attitudes towards LAI-PrEP and experiences of discrimination from healthcare providers are associated with reduced intentions to engage in conversations about starting LAI-PrEP. To effectively roll out LAI-PrEP among this population, targeted educational interventions are needed to address altitudinal concerns, particularly the long-term effects of the LAI-PrEP. Additionally, healthcare systems must prioritize strategies to reduce discrimination and enhance cultural competence to foster an environment where racial and sexual minority individuals feel supported and valued.
